# Non‐homology‐based prediction of gene functions in maize (*Zea mays* ssp. *mays*)

**DOI:** 10.1002/tpg2.20015

**Published:** 2020-04-29

**Authors:** Xiuru Dai, Zheng Xu, Zhikai Liang, Xiaoyu Tu, Silin Zhong, James C. Schnable, Pinghua Li

**Affiliations:** ^1^ State Key Laboratory of Crop Biology Shandong Agricultural University Taian 273100 China; ^2^ Quantitative Life Sciences Initiative, Center for Plant Science Innovation, and Department of Agronomy and Horticulture University of Nebraska‐Lincoln Lincoln NE 68588 USA; ^3^ Department of Mathematics and Statistics Wright State University Dayton OH 45435 USA; ^4^ State Key Laboratory of Agrobiotechnology, School of Life Sciences Chinese University of Hong Kong Hong Kong China

## Abstract

Advances in genome sequencing and annotation have eased the difficulty of identifying new gene sequences. Predicting the functions of these newly identified genes remains challenging. Genes descended from a common ancestral sequence are likely to have common functions. As a result, homology is widely used for gene function prediction. This means functional annotation errors also propagate from one species to another. Several approaches based on machine learning classification algorithms were evaluated for their ability to accurately predict gene function from non‐homology gene features. Among the eight supervised classification algorithms evaluated, random‐forest‐based prediction consistently provided the most accurate gene function prediction. Non‐homology‐based functional annotation provides complementary strengths to homology‐based annotation, with higher average performance in Biological Process GO terms, the domain where homology‐based functional annotation performs the worst, and weaker performance in Molecular Function GO terms, the domain where the accuracy of homology‐based functional annotation is highest. GO prediction models trained with homology‐based annotations were able to successfully predict annotations from a manually curated “gold standard” GO annotation set. Non‐homology‐based functional annotation based on machine learning may ultimately prove useful both as a method to assign predicted functions to orphan genes which lack functionally characterized homologs, and to identify and correct functional annotation errors which were propagated through homology‐based functional annotations.

**Abbreviations**
GWAS: genome wide association study
CDS: coding sequence
UTR: untranslated region
KB: kilobase
FPR: false positive rate
TP: true positive
FP: false positive
TN: true negative
FN: false negative
AUC-ROC: area under curve-receiver operator characteristic
PCA: Principal Component Analysis
RF: Random Forest
GBM: Gradient Boosting Machine
GLMNET: Lasso and Elastic-Net Regularized Generalized Linear Models
SVM: Support Vector Machines
PLS: Partial Least Squares
NNET: Neural Network
PMLR: Penalized Multinomial Logistic Regression
LDA: Linear Discriminant Analysis). IMP; inferred from mutant phenotype
EXP: inferred from experiment
IDA: inferred from direct assay
IPI: inferred from physical interaction
IGI: inferred from genetic interaction
IEP: inferred from expression profile
NAS: non-traceable author statement
TAS: traceable author statement
IC: inferred by curator
ND: no biological data available
HAD: inferred from high throughput direct assay
HEP: inferred from high throughput expression pattern). GO; gene ontology
ISS: inferred from sequence or structural similarity
ISM: inferred from sequence model
IBA: inferred from biological aspect of ancestor
IEA: inferred from electronic annotation
RCA: inferred from reviewed computational analysis

## 1 INTRODUCTION

The rapid acceleration in genome sequencing is providing complete sequences for dozens of new plant species each year (Chen et al., 2018; Michael & Jackson, 2013). Advances in both *de novo* and extrinsic evidence based gene structure annotation, combined with low cost and abundant RNA sequence datasets, aid the identification and definition of gene models across each new genome assembly (Campbell et al., 2014; Cook et al., 2019; Del Angel et al., 2018; Monnahan et al., 2019). However, while the accuracy and throughput of methods to define the structure of genes have grown rapidly, methods to experimentally determine the function of individual genes have not. Existing annotations are taken from a small set of proteins with direct experimental evidence and then these annotations are extrapolated to not only paralogous genes in the same genome but homologous genes—whether paralogous or orthologous—in the genomes of other species (Valencia, 2005). Among eukaryotes, fission yeast *Schizosaccharomyces pombe* has perhaps the most comprehensive set of functional gene annotations (Aslett & Wood, 2006). There are currently 41,912 gene associations for 5,397 gene products available on *Sz. pombe* GeneDB (Lock et al., 2018). Of these, 16,657 functional annotations for 2,302 genes (42.6% of 5,397 annotated genes) are directly derived from experiments, which include annotations with evidence codes IMP (inferred from mutant phenotype), EXP (inferred from experiment), IDA (inferred from direct assay), IPI (inferred from physical interaction), IGI (inferred from genetic interaction), and IEP (inferred from expression profile). Of those, a subset of 4,761 functional annotations for 1,459 genes (27.0% of all annotated gene models in *Sz. pombe*) are supported by mutant phenotype analysis (evidence code IMP). Among flowering plants, the model species *Arabidopsis thaliana* has been the subject of intensive and comprehensive genetic investigation. However, of the 28,775 annotated gene models in the TAIR10 *A. thaliana* reference genome, only 19.2% have functional annotations supported by mutant phenotypes (evidence code IMP) and 24.5% have functional annotations supported by other types of experimental evidence (e.g. IDA, IPI, IGI, IEP, HAD (inferred from high throughput direct assay) (inferred from high throughput direct assay), and HEP (inferred from high throughput expression pattern). An additional 30.4% of *A. thaliana* gene models are functionally annotated based on solely protein features, sequence similarity, or other forms of evidence which are used to infer homology. These include GO (gene ontology) terms supported by the evidence codes ISS (inferred from sequence or structural similarity), ISM (inferred from sequence model), IBA (inferred from biological aspect of ancestor), IEA (inferred from electronic annotation), and RCA (inferred from reviewed computational analysis). A quick aside on terminology. Homology refers to the state of two things sharing common ancestry. Sequence similarity is a type of evidence that two or more DNA or amino acid sequences share common ancestry. Here we chose to refer to methods which use sequence similarity to identify groups of genes that are apparently homologs, and propagate functional annotations between these approaches as homology‐based rather than sequence‐similarity‐based as we feel it more accuracy conveys the reasoning for this approach, that genes descended from a common ancestor are likely to have similar functions. An additional 19.7% of gene models are assigned functional annotations based only on evidence codes which are not directly linkable to evidence; NAS (non‐traceable author statement), TAS (traceable author statement), IC (inferred by curator), and ND (no biological data available). The final 6.2% of Arabidopsis gene models lack any functional annotation (Lamesch et al., 2011).

A significant challenge of homology‐based functional annotation is that these annotations are often propagated from one sequence to the next without associated data on provenance. Thus, it is often impossible or impractical to track a computationally assigned functional annotation back to the original source of experimental evidence. This presents a challenge, as mistaken findings related to protein functions will be published from time to time (Iyer et al., 2001), and once an experimentally derived functional annotation is assigned to homologs in other species, there is no way to “recall” this annotation. In fact, the annotation is likely to continue to propagate to new genome assemblies and to reannotations of existing assemblies (Brenner, 1999; Gilks, Audit, De Angelis, Tsoka, & Ouzounis, 2002, 2005; Valencia, 2005). It should be noted that this problem of error propagation is not present in all types of homology‐based functional annotations. In some cases, when a protein is annotated with a domain from the annotation of the domain in InterProScan or Pfam (Finn et al., 2015; Quevillon et al., 2005), it is indeed possible to trace back to what evidence was used to predict the function of the domain. Curated annotations made based on non‐sequence similarity evidence have been estimated to have an error rate of 13–18%, while curated annotations made based on sequence similarity evidence had an estimated error rate of 49% (Jones, Brown, & Baumann, 2007). In short, “functional annotations are propagated repeatedly from one sequence to the next, to the next, with no record made of the source of a given annotation, leading to a potential transitive catastrophe of erroneous annotations” (Karp, 1998). Homology‐based functional annotation also rests on the basic assumption that sequence similarity and functional similarity is highly correlated, which is an assumption that is not always correct as demonstrated by many cases of sub‐ and neo‐ functionalization between homologous genes (Brown, Gerlt, Seffernick, & Babbitt, 2006; Clark & Radivojac, 2011; Radivojac et al., 2013). Comparison between two yeast species (*Saccharomyces cerevisiae* and *Candida albicans*) identified numerous cases where homologous proteins appear to play different biological roles (Homann, Dea, Noble, & Johnson, 2009).

In addition to concerns with annotation accuracy, many species also contain a significant number of genes where homology‐based annotation is not possible. The genomes of *A.thaliana* and rice, respectively, are reported to contain 1,430 and 1,926 orphan genes which lack known homologs in other species (Guo, 2013; Guo, Li, Ling, & Ye, 2007). By definition, homology‐based methods are only able to make predictions when the function of at least one related sequence—whether detected through direct nucleotide or protein sequence similarity (Conesa et al., 2005), or more sensitive methods such as the presence of a shared protein domain or protein domain architecture (Finn et al., 2015; Hulo et al., 2006; Quevillon et al., 2005; Thomas et al., 2003)—has been experimentally characterized. As the result, genes belonging to orphan gene families and/or carrying only domains of unknown functions are likely to lack predicted or potential functions. This in turn contributes to the noted pattern of clustering of research efforts on more detailed characterization of genes with existing well characterized functions (Stoeger, Gerlach, Morimoto, & Amaral, 2018).

However, there exists a parallel set of non‐homology‐based approaches to predict the function of uncharacterized genes (Gabaldón & Huynen, 2004; Marcotte, 2000; Marcotte et al., 1999). Chromosomal context has been widely employed for functional prediction in prokaryotes where operons of genes involved in a single metabolic pathway or biological process are common (Edwards, Rison, Stoker, & Wernisch, 2005; Enault, Suhre, & Claverie, 2005). High rates of gene loss and horizontal gene transfer in prokaryotes can also be employed to assign predicted functions to genes with either similar or complementary phylogenetic distributions (Gaasterland & Ragan, 1998; Morett et al., 2003; Pellegrini, Marcotte, Thompson, Eisenberg, & Yeates, 1999). In eukaryotes such as maize and *A.thaliana*, mRNA co‐expression analysis has been shown to improve the prioritization of GWAS (genome wide association study) hits (Angelovici et al., 2017; Chan, Rowe, Corwin, Joseph, & Kliebenstein, 2011; Schaefer et al., 2018; Zheng et al., 2019). Protein co‐expression networks are also beginning to become more widely available and appear to capture different information content from mRNA co‐expression networks (Walley et al., 2016). Non‐homology‐based methods have been used to systematically develop functional predictions in prokaryotes and have been employed in yeast using topology of biological networks which are extended from protein–protein interaction for reconstruction of GO (Gligorijević, Janjić, & Pržulj, 2014). Furthermore, non‐homology‐based methods have been used to prioritize individual sets of candidate genes in plants (Angelovici et al., 2017; Chan et al., 2011; Schaefer et al., 2018; Zheng et al., 2019). However, genome‐wide functional annotation in plants still relies primarily on homology‐based methods.

**Core Ideas**

- The functions of genes can be predicted without homology data
- Non‐homology methods work better for predicting the biological role of proteins
- Better data on the sources of existing gene functional annotations are needed

Here we sought to evaluate the potential of using various supervised classification algorithms to predict the function of annotated genes in the absence of homology data, but instead using a range of molecular, structural, and chromatin data types. If successful, accurate prediction of gene function from these data types would have a number of complementary strengths to current approaches to gene function annotation. As described above, there may be incorrect gene function annotations which have propagated from database to database, and an independent method to assess gene function could highlight cases where existing functional annotations should be rechecked by an expert human annotator. In addition, because when molecular, structural, and chromatin data are available at all, they are frequently available for all or nearly all annotated gene models, predictions of gene function based on these features would aid in hypotheses generation for orphan genes and suggest experimental approaches to validating the function of more genes which lack experimentally characterized homologs. This initial analysis focused on maize (*Zea mays* ssp. *mays*), a widely studied genetic model and economically vital crop species. The need for non‐homology‐based functional annotations is pressing in maize, particularly as there is evidence these new and variably present genes may be involved in hybrid vigor (Baldauf, Marcon, Paschold, & Hochholdinger, 2018, 2016; Paschold et al., 2014). Maize has an extensive collection of functional genomic datasets, including large RNA and protein expression atlases (Stelpflug et al., 2016; Walley et al., 2016), methylation and histone modification profiling datasets (Dong et al., 2017), one of the largest collection of characterized and cloned loss‐of‐function mutants of any plant species (Oellrich et al., 2015; Schnable & Freeling, 2011) and these data sets were used as the features from which a set of eight supervised classification algorithms were trained to predict gene function. In this project, we evaluated the potential for using supervised machine‐learning‐based classification algorithms to predict the function of annotated maize genes using purely non‐homology‐based features, and seek to determine which kinds of molecular, structural, or chromatin features are likely to be more or less beneficial additions when estimating gene function using algorithms of this type.

## 2 METHODS

### 2.1 Composition of the prediction variable dataset

Predictive variables were divided into six categories: Gene Model Structure, RNA Expression, Protein Expression, Chromatin, Co‐Expression, and Population Genetics. Gene structural features included gene length from transcription start site to transcription stop site, including introns, exon number, coding sequence length, 3′ UTR (untranslated region) and 5′ UTR length. These values were calculated for each gene using the published AGPv4 maize genome sequence and annotation (Jiao et al., 2017). Nucleotide composition and the GC content were calculated using all sequence from the annotated transcription start site to the annotated transcription stop site.

For protein‐coding genes, a codon usage bias score which describes the degree of bias towards the most frequently used codons for multiple encoding amino acids in a given species was calculated following the method described in (Sharp & Li, 1987) as implemented in the SeqIO module in biopython (v1.72) package (Cock et al., 2009).

The initial set of RNA expression features included data from 2–3 replicates of 79 distinct tissue types in the maize inbred B73 (222 total samples) (Stelpflug et al., 2016) and 52 samples from biotic and biotic stress studies of B73 in different labs (Makarevitch et al., 2015; Opitz et al., 2014; Swart et al., 2017) for a total of 274 distinct samples. Normalized (FPKM: fragments per kilobase of exon per million aligned reads) expression values for each gene in each experiment were obtained from (Hoopes et al., 2019).

Protein expression features consisted of normalized protein abundance data quantified in dNSAF (distributed normalized spectral abundance factor) for 33 distinct tissues sampled from B73 were obtained from (Walley et al., 2016). B73 AGPv2 gene models were converted to B73 AGPv4 using a conversion list published on MaizeGDB (Portwood et al., 2018).

Chromatin features included DNA methylation (quantified separately in CG, CHG, and CHH contexts), three histone modifications (H3K4me3, H3K27me3, H3K27ac), and open chromatin as quantified by ATAC‐seq. Raw sequence data for bisulfite‐seq, ChIP‐seq for H3K4me3, H3K27me3, and H3K27ac histone modifications, and ATAC‐seq was downloaded from PRJNA391551 in the NCBI SRA (Dong et al., 2017). DNA methylation was quantified using Bismark (v0.19) with parameters “‐L 50, ‐N 1” (Krueger & Andrews, 2011). ATAC‐seq and histone ChIP‐seq reads were aligned to AGPv4 of the maize reference genome using gsnap (v2018‐03‐25) (Wu, Reeder, Lawrence, Becker, & Brauer, 2016) with parameters “‐m 0.02, ‐B 5, ‐n 1, ‐Q, –nofails”. Alignment files were then used to call peaks using the protocol previously described in (Dong et al., 2017).

For each of these chromatin features, scores were calculated for three regions: one using the gene body, defined as the region from the annotated transcription start site to the annotated transcription stop site, a second for the upstream region, defined as a 2 KB (kilobase) region directly upstream of the transcription start site, and a third for the downstream region, defined as the 2 KB region directly downstream of the transcription stop site. For each BS‐seq dataset, for each of the three regions relative to each gene and each of three methylation contexts (CG, CHG, CHH), a single percentage score was calculated. These percentages were calculated as the ratio of all cytosines in that context in that genomic interval which were classified as “methylated” (≥5 mapped reads and with *>*50% of mapped reads showing methylation) to the total number of cytosines in that context in that genomic interval. For each ChIP‐seq and ATAC‐seq dataset, two features were calculated for each genomic interval: the maximum intensity among peaks overlapping that interval and the proportion of that interval covered by peaks using methods previously described in (Lloyd, Tsai, Sowers, Panchy, & Shiu, 2017).

The co‐expression set of features consisted of 12 binary variables defining membership in each of the 12 co‐expression models defined by (Hoopes et al., 2019).

For natural diversity features, raw genotype calls of 277 resequenced inbreds in maize 282 association panel (Bukowski et al., 2017) were downloaded from Panzea (https://www.panzea.org/). Only biallelic SNPs were considered as variations in the given population for this study. SNP filtering, imputation and assignment to maize AGPv4 gene body region was processed as a previous study (Liang, Qiu, & Schnable, 2019). SNP number per gene was determined by the number of final detected SNPs per AGPv4 gene.

### 2.2 Dimension reduction

Principal‐component‐based dimension reduction was evaluated for RNA abundance and protein abundance data using R prcomp() function with parameters “center = TRUE, scale = TRUE”. For each set of features, 50 principal components were calculated. In each case, the decision on how many principal components to include was based on the cumulative proportion of variance explained.

### 2.3 Defining the subset of gene models and functional annotations

Several important features like protein abundance data for maize vegetative and reproductive stages, are only available for maize AGPv2. As the result, we constrained this analysis and only considered a set gene models which had a 1:1 relationship between a single gene model in the maize reference genome version AGPv2 and a single gene model in the maize reference genome version AGPv4 (Liang et al., 2019). A small number of genes with missing values for more than half of the total set of 369 features were omitted from subsequent analyses. For the remaining genes, features were centered, scaled and imputed (for missing values) using preProcess() function in caret (v6.0‐80) R package (Kuhn, 2015).

An implicit GO term assignment can occur when a specific GO term is explicitly assigned to a gene, each parent of that GO term is also implicitly assigned to the same gene. The parents() function in goatools (v0.8.9) python package was used to add the implicit GO terms to each gene (Klopfenstein et al., 2018). After explicitly assigning implicit GO annotations to genes, GO terms which were assigned to less than 100 genes or more than 5,000 gene models were excluded.

### 2.4 Implementing machine learning algorithms

The eight machine learning algorithms, i.e. random forest, neural network, svmRadial, glmnet, lda, penalized multinomial regression, partial least squares and gbm with parameter “tuneLength = 5”, evaluated as part of this study were all implemented in the R package caret (v6.0‐80) (Kuhn, 2015). For each GO term, a balanced training data was constructed using the set of maize genes assigned with that annotation as the “positive” set and a randomly selected equal number of genes not assigned with that annotation as the “negative” set. A 20% of the negative and positive genes from each training set were set aside as the hold‐out testing data to assess model performance. The remaining 80% of data was used to train each algorithm for each GO term. A 10‐fold cross validation was used. The three stacking ensemble methods evaluated in our study were also tested using implementations in the caret package (Kuhn, 2015). Each of the three was employed as a supervisor model and was provided with the output of three primary predictive methods (random forest, gbm, and glm) with “tuneLength = 3”. R source code used to conduct all GO prediction analyses in this paper has been deposited online (https://github.com/xiuru/Prediction-of-Gene-Functions-in-maize).

### 2.5 Evaluating prediction accuracy

Accuracy, FPR (false positive rate), recall, precision, F1 score, AUC‐ROC (Area Under Curve‐Receiver Operator Curve), and consistency score were calculated for each GO term. Accuracy = (TP+TN)/(TP+TN+FP+FN) where TP, true positive; FP, false positive; TN, true negative; FN, false negative). The FPR was calculated as the ratio between the number of negative events wrongly categorized as positive and the total number of actual negative events (FP/FP+TN). Recall was defined as the fraction of positive instances that have been retrieved over the total amount of positive instances (TP/TP+FN). Precision was defined as the fraction of positive instances among the retrieved instances (TP/TP+FP). The F1 score was calculated as the harmonic mean of precision and recall. AUC‐ROC was calculated as the ratio of the total the area under the plot of receiver operating characteristic curve, to the total area contained within the plot. For permutation testing to evaluate the potential of over‐fitting, the same training and testing datasets were used, and the same algorithms employed, but genes were shuffled between the positive and negative categories. A manually reviewed gold standard annotation set was downloaded from Cyverse (https://doi.org/10.7946/P2S62P) to evaluate prediction accuracy on manually annotated genes.

## 3 RESULTS

We assembled a set of descriptors for each gene, including gene structure, population genetics, expression, histone modification and DNA methylation features (Supplemental Table S1). This dataset included features calculated from the alignment of sequence reads to the maize AGPv4 reference genome and data mined from previously published papers (Bukowski et al., 2017; Dong et al., 2017; Hoopes et al., 2019; Jiao et al., 2017; Liang et al., 2019; Walley et al., 2016). Some important features, for example, protein abundance data for maize vegetative and reproductive stages, are only available for prior versions of the maize reference genome. As the result, we constrained this analysis and only considered a set of 29,428 gene models which had a 1:1 relationship between a single gene model in the maize reference genome version AGPv2 and a single gene model in the maize reference genome version AGPv4 (Liang et al., 2019; Schnable et al., 2009; Wang et al., 2016). Many algorithms for making predictions from input feature sets are intolerant of missing values. While the overall rate of missing data for this 1:1 gene set was low, a small number of genes (1,995 genes) have missing values for more than half of the total set of 369 features (Supplemental Figure S1). These genes were omitted from subsequent analyses. For the remaining 27,433 genes, missing values were imputed using the median value for that feature across all the genes where that feature was successfully scored.

### 3.1 Potential for dimensional reduction among non‐homology features

**FIGURE 1:**
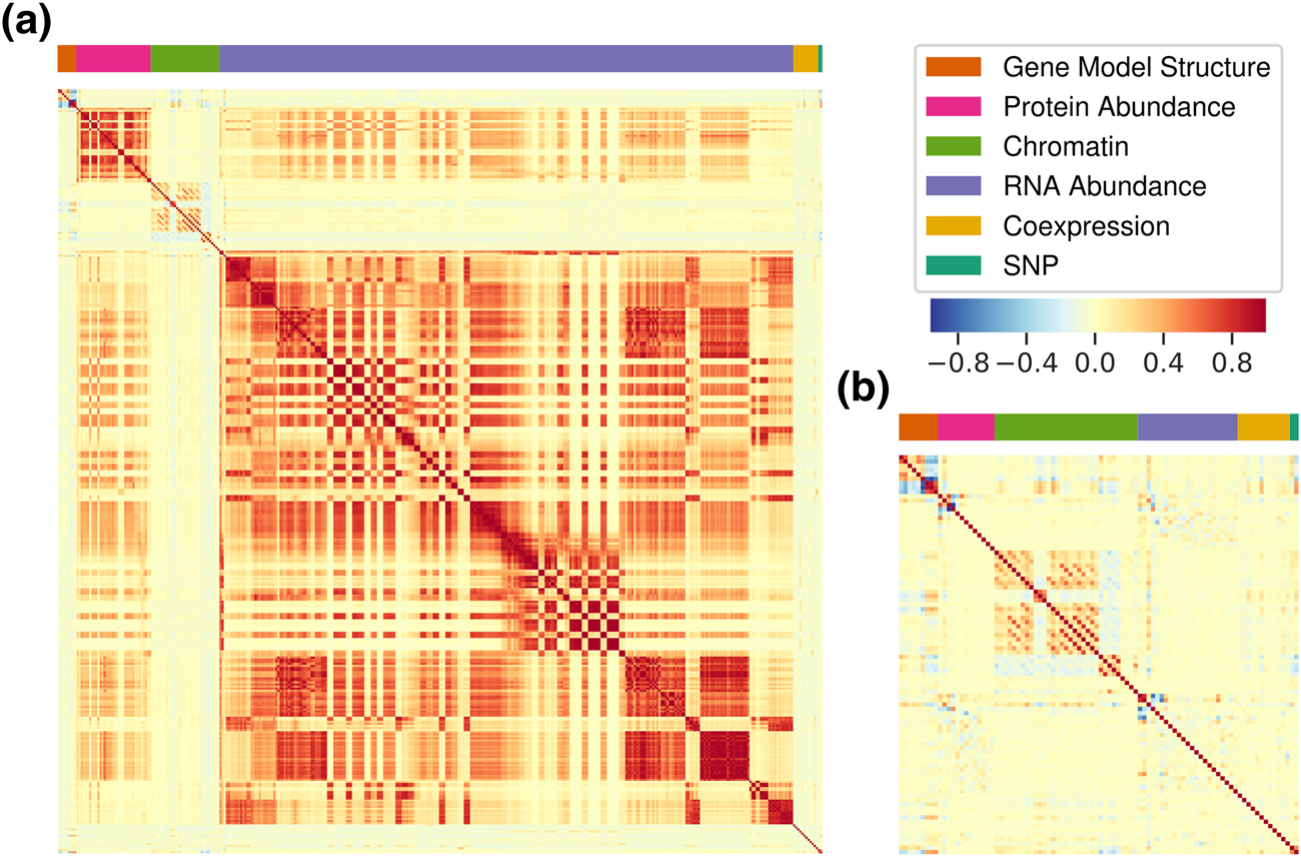
Correlations among different features in the prediction dataset. (a) Spearman correlations and group membership among all 369 original features. (b) Spearman correlations and group membership for 92 features remaining after targeted principal‐component‐based dimension expression data. The ordering of individual features from top to bottom and left to right is provided in Supplemental Table S2

Spearman correlation coefficients were calculated among the 369 features (Figure 1). The two largest classes of features, i.e. RNA abundance (274 features) and protein abundance (33 features), showed substantial between‐feature correlation. Supervised machine learning classification models tend to overfit when trained with excessively large numbers of features. This overfitting decreases predictive performance on non‐training datasets. Dimensional reduction techniques seek to address this problem by reducing the total number of features available for training without significantly reducing the overall information content of the dataset. Dimensional reduction algorithms can generally be divided into the categories of feature extraction and feature selection. Principal Component Analysis (PCA) is a widely used feature extraction technique that improves learning performance, reduces computational complexity, builds better models and decreases the required memory space (Tang, Alelyani, & Liu, 2014). More than 90% of the variance in RNA abundance and protein abundance could be captured by 20 and 10 principal components respectively (Supplemental Figure S2). These principal components were used in place of the original RNA and protein abundance features, which decreased the number of possible predictive variables from 369 to 92. This decrease also substantially reduced the degree of correlations between possible predictive variables (Figure 1). The 95th percentile and 99th percentile for the absolute values of Spearman correlation (r_s_) dropped from 0.76 and 0.94 to 0.22 and 0.51, respectively (Figure 1b).

Classifiers were trained using either all 369 predictor features, the reduced set of 92 predictor features remaining after targeted dimensional reduction, or 50 predictor features extracted from untargeted dimensional reduction for complete set of 369 features. Across random forest models trained of each of the 1,562 GO terms in this analysis, those trained with the full set of 369 features exhibited prediction accuracies of 0.35 to 0.93 with a median of 0.67. Models trained using the reduced set of 92 predictor features exhibited prediction accuracies of 0.41 to 0.93 with a median of 0.68. Models trained using 50 untargeted principal components exhibited prediction accuracies of 0.42 to 0.86 with a median of 0.63. The increase in prediction accuracy for the targeted dimensional reduction models relative to the full models was statistically significant although the effect size was modest (*p* = 0.0002; two tailed paired t‐test). While targeted dimensional reduction increased prediction accuracy, untargeted dimensional reduction did not. Models trained using a set of 50 principal components extracted from the complete set of 369 features provided significantly lower prediction accuracy than either the total feature set (*p* = 5.96× 10^−158^; two tailed paired t‐test) or the targeted dimensional reduction feature set (*p* = 3.89 × 10^−192^; two tailed paired t‐test) (Supplemental Figure S3).

Prediction accuracy was evaluated using shuffled data to test whether, even after dimension reduction, over fitting might be occurring. Prediction accuracy for individual GO terms ranged from 0.47 to 0.57 with a median of 0.51, only slightly higher than expected of random predictions. The median prediction accuracy of 0.51 for shuffled GO term assignments was consistent across 4 sequential permutations of the data. As targeted dimensional reduction increased prediction accuracy to a modest extent while retaining the ability to evaluate segregated models using only specific biological data types requiring different sets of procedures to assay in new species, the dataset generated using targeted dimensional reduction was employed for downstream analyses.

Multiple distinct sets of GO predictions exist for the maize reference genome (Goodstein et al., 2011; Tello‐Ruiz et al., 2015; Wimalanathan, Friedberg, Andorf, & Lawrence‐Dill, 2018). We chose to use the maize GAMER dataset as our starting point for training and evaluating non‐homology‐based prediction algorithms (Wimalanathan et al., 2018). The published maize GAMER dataset includes 9,336 GO terms which are directly assigned to one or more gene models, and an additional 2,757 GO terms which are implicitly assigned to one or more gene models. An implicit GO term assignment can occur when a specific GO term is explicitly assigned to a gene. In this case, each parent of that GO term is also assigned to the same gene implicitly. We utilized both implicit and explicit GO term assignments. The initial dataset thus consisted of 12,093 GO terms and each go term was assigned to one or more of the 39,324 gene models in the B73 AGPv4 maize reference genome. We chose to exclude both extremely common GO terms (e.g. GO:0008150 “Biological Process”) and extremely rare GO terms. Extremely common GO terms tend to be low information content. Extremely rare GO terms are unlikely to possess enough known positive genes to accurately train prediction algorithms. After excluding GO terms assigned to *<*100 genes or *>*5,000 genes in our set of 27,433 genes with feature data, 1,562 GO terms—including 1,148 Biological Process, 151 Cellular Component and 263 Molecular Function terms—remained for downstream analyses.

#### 3.1.1 Selection of random forest for gene function prediction

**FIGURE 2:**
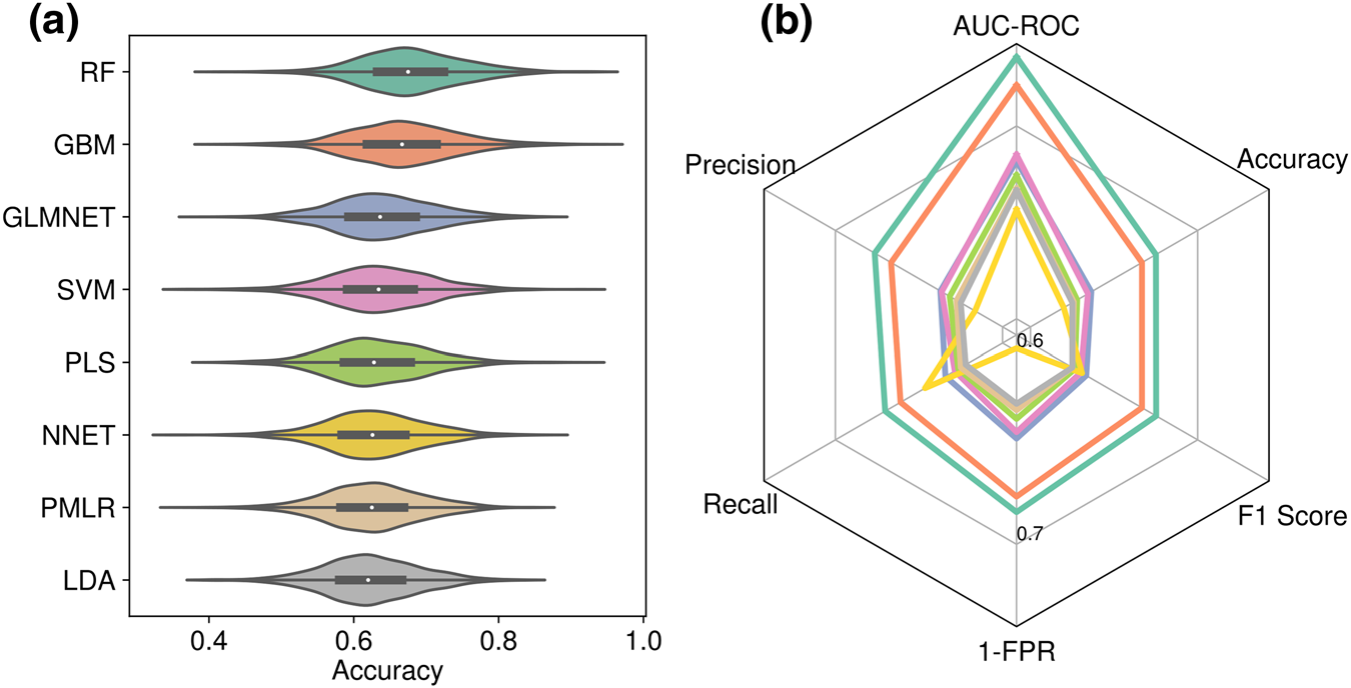
Performance of eight machine‐learning‐based supervised classification algorithms in predicting gene functions using non‐homology‐based predictor variables. (a) Distribution of prediction accuracies for 1,562 GO terms using 8 methods. RF (Random Forest); GBM (Gradient Boosting Machine); GLMNET (Lasso and Elastic‐Net Regularized Generalized Linear Models); SVM (Support Vector Machines with Radial Basis Function Kernel); PLS (Partial Least Squares); NNET (Neural Network); PMLR (Penalized Multinomial Logistic Regression); LDA (Linear Discriminant Analysis). (b) Median values for each of the eight algorithms. Color labeling in panel B correspond to the color labeling of each algorithm in panel A

Eight machine‐learning‐based supervised classification algorithms including random forest (Liaw & Wiener, 2002), stochastic gradient boosting machines (Ridgeway, Southworth, & RUnit, 2013), Lasso and Elastic‐Net Regularized Generalized Linear Models (Friedman, Hastie, & Tibshirani, 2010; Simon, Friedman, Hastie, & Tibshirani, 2011), Support Vector Machines with Radial Basis Function Kernel (Karatzoglou, Smola, Hornik, & Zeileis, 2018, 2004), partial least squares (Wehrens & Mevik, 2007), neural network (Ripley, Venables, & Ripley, 2016; Venables & Ripley, 2002), penalized multinomial regression (Ripley et al., 2016; Venables & Ripley, 2002), and linear discriminant analysis (Ripley et al., 2013; Venables & Ripley, 2002) were evaluated for their accuracy in predicting GO annotations. Benchmark genes for every GO term were divided into the sets of 80% training and 20% testing. For training data, 10‐fold cross validation was performed for all machine learning methods. Validation accuracy and testing accuracy, i.e. the accuracy in testing dataset, were both calculated for the 8 algorithms and comparisons of the algorithms were based on the accuracy in testing dataset. Based on the average accuracy across all GO terms tested, random forest and gbm methods performed the best and second best respectively (Figure 2a‐b). Random forest was the best performing algorithm for 52% of all GO terms (average rank from 1–8 = 2.0), and gbm was the best performing algorithm for 30% of GO terms (average rank from 1–8 = 2.7). No other algorithm had an average rank <4 or was the performing algorithm for >8% of tested GO terms. This ranking was consistent across sets of GO terms with different annotation frequencies, as well as for GO terms within each of the three

The GO domains include: Biological Process, Cellular Component, Molecular Function (Supplemental Table S3). This ranking was also consistent when performance was calculated in different ways. Random forest exhibited the best performance based on calculations of precision (proportion of predicted genes that are truly positive), recall (proportion of true positive genes recovered), F‐measure (harmonic mean of precision and recall), consistency score, and AUC‐ROC (Figure 2b). Ensemble methods were also evaluated however these did not show a significant increase in prediction accuracy compared to pure random‐forest‐based prediction (Supplemental Figure S4).

### 3.2 Higher prediction accuracy for biological process GO terms

**FIGURE 3:**
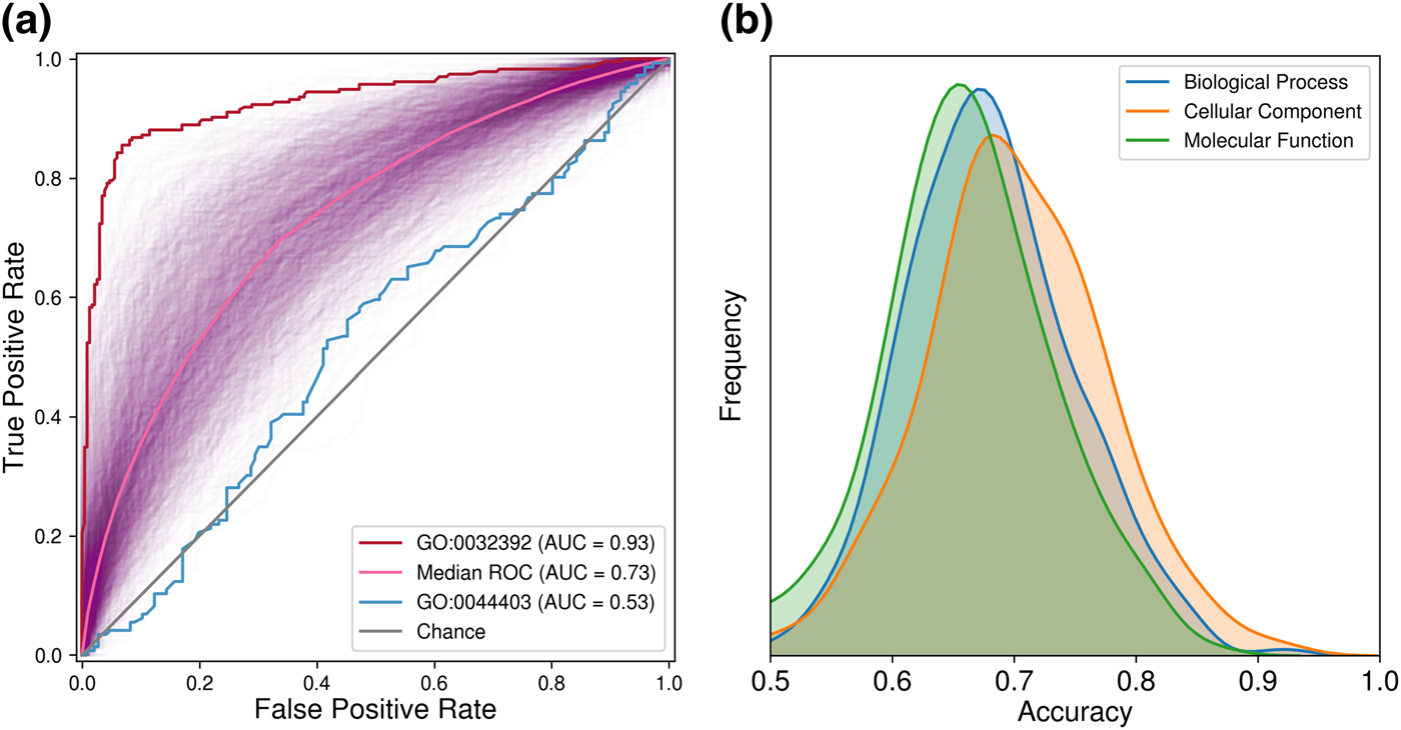
Prediction accuracy for individual GO terms varies in response to different characteristics of those terms. (a) Distribution of AUC‐ROC values for random‐forest‐based prediction of 1,562 GO terms, including information on the single best and second worst performing GO terms based on AUC‐ROC GO:0032392 (DNA geometric change; Biological Process) and GO:0044403 (Symbiotic Process; Biological Process). The worst performing GO term (GO:005877) was for a biological process GO term which does not occur in plants. (b) Distribution of prediction accuracies for individual GO terms in the Biological Process, Cellular Component and Molecular Function domains using random‐forest‐based prediction

ROC (receiver operating characteristic) curves for the prediction accuracy of random‐forest‐based prediction—determined from 10‐fold cross validation—were plotted for individual GO terms (Figure 3a). Details for the performance measures of every GO term provided in Supplemental Table S4. As a control, AUC‐ROC values were also calculated for genes with shuffled functional annotations. The 5th and 95th percentile of AUC‐ROC values from 4 times of gene label shuffling for individual GO terms were 0.45 and 0.56 and for multiple iterations the median is 0.51. These values were consistent with expectations for random labeling of balanced data.

Random forest testing accuracy for individual GO terms ranged from 0.41 to 0.93 with a median of 0.68. The single best performing GO term prediction model, assessed based on accuracy, was for GO:0006270 (DNA replication initiation) using random forest (precision = 95.2%, recall = 90.9%, FPR = 4.5%, Accuracy = 0.93, AUC‐ROC = 0.92, Consistency score = 0.87). The GO terms related to DNA replication (GO:0006270, DNA replication initiation, Accu = 0.93), modification (GO:0016556, mRNA modification, Accu = 0.90; GO:0006304, DNA modification, Accu = 0.85), methylation (GO:0006346, methylation‐dependent chromatin silencing, Accu = 0.93; GO:0001510, RNA methylation, Accu = 0.91) and metabolic process (GO:0009220, pyrimidine ribonucleotide biosynthetic process, Accu = 0.86) are well predicted using non‐homology features. On the other end of the distribution, examples of GO terms with the low prediction accuracy were (GO:0022832, voltage‐gated channel activity, Accu = 0.48; GO:0005216, ion channel activity, Accu = 0.48) and regulation of a process (GO:0050778, positive regulation of immune response, Accu = 0.52; GO:0051348, negative regulation of transferase activity). GO terms with higher prediction accuracy were drawn primarily from the Biological Process domain while GO terms with the lowest prediction accuracy belonged primarily to the Molecular Function domain.

To test whether this finding represented a consistent pattern, the distribution of prediction accuracies was evaluated separately for GO terms belonging to each of the three domains (Biological Process, Cellular Component, and Molecular Function). GO terms involved in Cellular Component have the highest median accuracy (Figure 3b). Cellular Component GO terms were the rarest of the three domains (151 GO terms of 1,562 total terms tested). Median accuracy for Biological Process GO terms was modestly lower than for Cellular Component. Biological Process GO terms were much more abundant (73%) in 1,562 GO terms test, which may explain why the most accurate individual GO terms were drawn from this domain. GO terms from the Molecular Function domain had the lowest median accuracy, and there were many Molecular Function GO terms, particularly those related to channel, transporter, enzyme activity or binding with extremely low accuracy (Supplemental Table S4). This ranking of accuracy across GO domains was largely consistent across GO terms with different population sizes of genes carrying the annotation and across the results from predicting using different machine learning algorithms (Supplemental Table S3).

### 3.3 Contribution of different feature types to prediction accuracy

**FIGURE 4:**
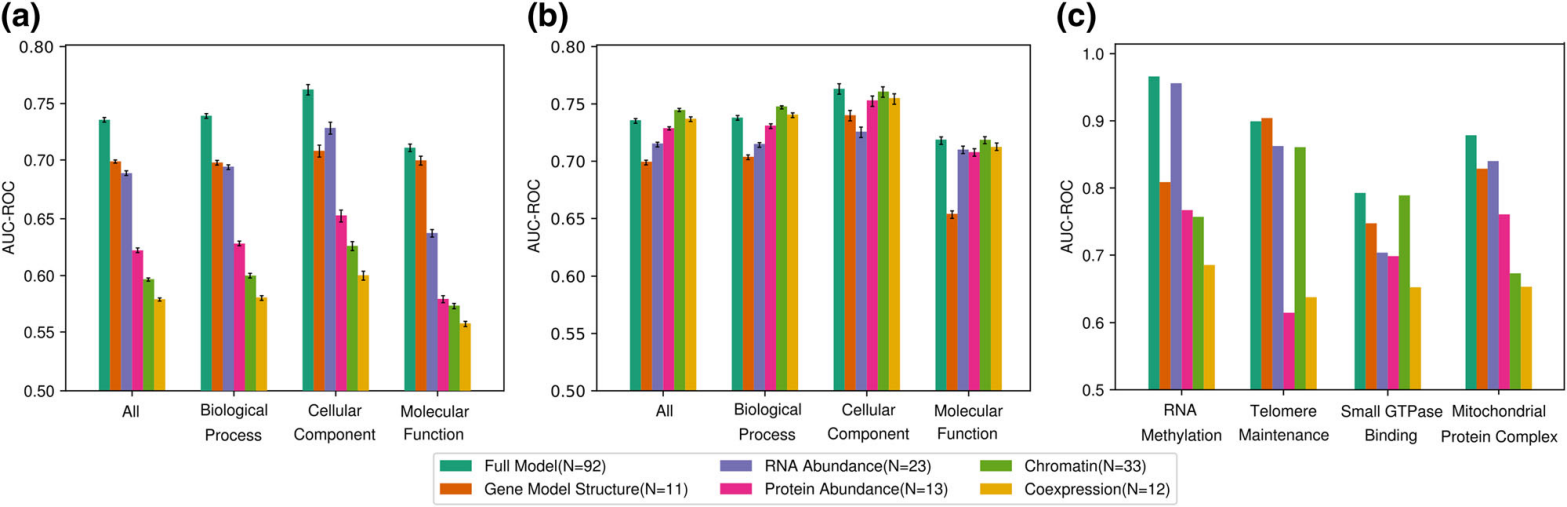
Contributions of each of five types of features to the overall functional prediction accuracy. (a) Median AUC‐ROC values for all GO terms, and GO terms classified based on domain for the complete model, and partial models trained using only RNA abundance features, only chromatin features, only gene structure futures (including 2 population genetic features), only protein abundance features, or only co‐expression features. Error bars indicate Standard Error around the median AUC‐ROC calculated from the individual prediction accuracies for all 1,562 GO terms or every GO term in 3 GO domains. (b) AUC‐ROC values for models constructed using data for four out of five feature types. Bigger decreases relative to the full model indicate feature types which provide larger amounts of non‐redundant information for GO prediction. Error bars indicate Standard Error around the median AUC‐ROC among all tested GO terms within a given domain. (c) Examples of the same comparison shown in panel A for individual GO terms: RNA Methylation (GO:0001510) (Biological Process); Telomere Maintenance (GO:0000723) (Biological Process); Small GTPase Binding (GO:0031267) (Molecular Function); Mitochondrial Protein Complex (GO:0098798) (Cellular Component). As this panel displays data for individual GO terms each bar represents a single value, not a median, and no error bars are show

Separate predictions were conducted using distinct subsets of features to assess relative contributions of different types of features to the overall accuracy of non‐homology‐based functional prediction by building different machine learning models using random forest algorithms. The ranking of prediction accuracy was largely consistent across the three primary GO term domains: Biological Process, Cellular Component, and Molecular Function. Models trained using only gene model structure features or trained using only RNA expression features provided approximately equal independent prediction accuracy. One exception was in the Molecular Function domain where models trained using only gene structure features performed almost equivalently to the complete model (median AUC‐ROC = 0.69 and 0.70 for the models using structural data only and full models, respectively). Models for predicting Molecular Function GO terms trained using only RNA expression features performed significantly worse than the complete model. Models trained using only chromatin features or only co‐expression features did not perform well in any of the three domains (Figure 4a). Excluding chromatin features increased will increase the prediction accuracy for both the specific set of Biological Process GO terms as well as the complete population of tested GO terms, while gene model structure and RNA abundance appear to provide distinct and partially non‐redundant contributions to prediction accuracy of both the Biological Process and Cellular Component GO term populations. While it was possible to obtain models with some prediction accuracy using only chromatin or coexpression features, models which excluded these features performed equally to the full model, suggesting the information content of these feature types is likely to be independently by other feature types (Figure 4). In addition, the importance of each of the 92 individual feature across the 1,562 GO terms was calculated using the using caret varImp and provided as Supplemental Table S5. At the level of individual GO terms, there were a number of GO terms where models trained using only protein expression features (99 GO terms 6.3%) or chromatin state features (35 GO terms 2.2%) had better performance than any of the other component models (Figure 4c; Supplemental Table S6). In a minor number of cases (15 GO terms 0.96%) the model trained using only co‐expression features provided the highest accuracy of any of the component models (Supplemental Table S6).

### 3.4 Evaluation using manually reviewed annotations

Prediction accuracy was independently evaluated using a set of 476 GO terms assigned to 1,619 gene models by manual curation of direct assay and mutant phenotype evidence (Monaco et al., 2013; Wimalanathan et al., 2018). From this set, 263 GO terms overlapped with the 1,562 GO terms for which prediction models were trained, and 21 of the 263 overlapping GO terms were assigned to more than 10 genes in the gold standard set. New models were trained for each of these 21 GO terms using GAMER training data with all gold standard genes masked. Twenty of the 21 GO terms achieved a prediction accuracy >0.75 with a median value of 0.83.

## 4 DISCUSSION

Accurate and precise annotation of gene model functions in the absence of gene‐by‐gene genetic analysis remains challenging. In most species, the vast majority of genes have not been studied or characterized directly. Instead, when functional annotations are present, they are drawn from functional characterization of homologous genes. Homology‐based approaches may also introduce erroneous and misleading functional annotations. Firstly, genes which are homologous will not always perform the same biological function or be localized to the same cellular compartments. For example, R2R3‐MYB transcription factors are all homologous to each other yet play different roles regulating responses to multiple stress conditions, controlling plant development and cell fate, or regulating secondary metabolism (Du, Feng, Yang, Huang, & Tang, 2012). Secondly, because homology‐based functional annotations are often drawn from datasets and databases which were originally also annotated based on homology, it is possible for incorrect functional annotations to propagate through biological databases indefinitely. Estimates of the mis‐annotation using experimentally well‐characterized sets of enzymes can range from about 25% to over 60% (Schnoes, Brown, Dodevski, & Babbitt, 2009). Finally, 5 to 15% of annotated gene models in the genomes of many species are “orphans” without detectable homology to any protein with a characterized function. Here we sought to evaluate whether using machine learning methods and a set of non‐homology‐based features can complement existing methods for functional annotation. Non‐homology‐based methods may ultimately be able to correctly assign new functional annotations to gene models and identify potentially inaccurate existing functional annotations.

It is important to discuss one critical limitation of the analyses employed here. While non‐homology‐based annotation approaches ultimately hold the potential to identify and correct errors introduced by homology‐based annotation, in this study a set of functional annotations derived from homology‐based annotation were treated as ground truth. As the result, the true recall of non‐homology‐based methods may be higher than the estimated recall in this study, as some false negatives may in fact represent errors in the underlying functional annotations. Going forward, there is a clear need for curated sets of experimentally supported functional annotations for maize equivalent to those previously generated for species such as yeast and *A. thaliana* (Aslett & Wood, 2006; Lamesch et al., 2011). However, based on testing using a modest number of existing manually curated GO term assignments in maize, it appears that prediction models trained on homology‐based annotations may indeed achieve significant prediction accuracy when evaluated using functional annotations derived from direct evidence. It should also be noted that randomly splitting data into training and testing sets can tend to overestimate prediction accuracy in the real world, where systematic differences between training and prediction datasets can be more common (Sheridan, 2013). Models trained using functional annotations currently assigned to only one or several homologous gene families may learn signatures of those gene families rather than the annotated function itself. Gene family guided splitting of training and testing datasets is one potential method which could be used to control for this potential confounding variable (Washburn et al., 2019). However, there are also some reasons to be optimistic. For example, in this study each GO term was treated as a discrete unit. Accuracy metrics which leverage the relationships between GO terms would provide ways to evaluate the accuracy of classifiers in a more nuanced fashion that would capture “near miss” annotations (Plyusnin et al., 2018). For example a gene which should be assigned GO:0019685 (“photosynthesis, dark reaction”) and is instead assigned GO:0019684 (“photosynthesis, light reaction”) provides partially correct information as the two GO terms share a common parent one step up in the directed acyclic graph of GO relationships. While acknowledging these limitations and necessary future steps some intriguing initial patterns are still apparent in this initial trial of non‐homology‐based function annotation.

Machine‐learning‐based functional annotation showed strengths which are complementary to known accuracy patterns of primarily homology‐based methods. Specifically, homology‐based functional annotation has been reported to show higher accuracy for GO terms in the Molecular Function domain (Jiang et al., 2016; Radivojac et al., 2013). In contrast, we found that non‐homology‐based predictions exhibited the highest prediction accuracy in the Cellular Component and Biological Process domains, and the lowest accuracy in Molecular Function (Figure 3b). Molecular functions (e.g. transcription factors, transporters, structural proteins) are likely to be conserved between homologous sequences. In contrast, the cellular localization and biological role of a given transcription factor or signal transduction component can vary and diverge substantially between even closely related homologs (Du et al., 2012). Genes involved in the same biological process or localized to a specific cellular compartment may be more likely to exhibit shared features such as co‐expression than specific classes of transcription factors or transporters which may be localized to different cell types or expressed only in response to different environmental stimuli.

Going forward there are a number of potential avenues to improve the accuracy of genome‐wide non‐homology‐based functional annotation. As discussed above, the incorporation of more detailed provenance information for existing functional annotations will serve both to train more accurate models, and to more accurately quantify the performance of these models. There are also additional types of non‐homology‐based predictive variables which could be incorporated in the future. These include more extensive protein and mRNA expression data, particularly from different stress conditions, experimentally derived protein‐protein interaction data, descriptors of population genetic features including different types of selection and diversity, and as well as incorporating the results of quantitative genetic analyses using different types of phenotypes in different environments. Two challenges for future studies are how to integrate these heterogeneous data sources and how to deal with incomplete and noisy data.

## Supporting information

Table S1: Full set of predictor variable values employed in this study.

Table S2: Names of features used in this study.

Table S3: Detailed performance metrics for different classification methods when employed GO terms drawn from different domains.

Table S4: Performance evaluation metrics for individual GO terms.

Table S5: Importance of different predictor variable types for individual GO terms.

Supporting Material

Supporting Material

Supporting Material

Supporting Material
